# Risk factors associated with beta-peripapillary atrophy in individuals of African ancestry with primary open-angle glaucoma

**DOI:** 10.1038/s41433-025-03988-8

**Published:** 2025-10-07

**Authors:** Tzuriel Sapir, Rebecca Salowe, Yineng Chen, Yan Zhu, Roy Lee, Ebenezer Daniel, Prithvi Sankar, Victoria Addis, Eydie Miller, Gui-Shuang Ying, Joan M. O’Brien

**Affiliations:** 1 Center for Genetics of Complex Disease, Ophthalmology Department, Perelman School of Medicine, University of Pennsylvania, Philadelphia, PA USA; 2 Center for Preventive Ophthalmology and Biostatistics, Perelman School of Medicine at the University of Pennsylvania, Philadelphia, PA USA

**Keywords:** Risk factors, Population genetics, Prognostic markers, Optic nerve diseases, Epidemiology

## Abstract

**Background:**

Beta-Peripapillary Atrophy (beta-PPA) is an optic nerve head change associated with primary open-angle glaucoma (POAG). We evaluated demographic, ocular, and genetic risk factors for beta-PPA in individuals of African ancestry with POAG.

**Methods:**

POAG cases were recruited from the Primary Open-Angle African American Glaucoma Genetics (POAAGG) study. Beta-PPA was defined as hypopigmentation with visible choroidal vessels and sclera adjacent to the optic disc. Univariable and multivariable regression models assessed risk factors for the presence and extent of beta-PPA.

**Results:**

Among 3381 eyes, 969 (28.6%) had beta-PPA. Beta-PPA was associated with older age (OR = 2.90, *p* < 0.001) and larger vertical cup-to-disc ratio (CDR) (OR = 1.58, *p* = 0.007), and was less common in females (OR = 0.53, *p* < 0.001). Beta-PPA presence was negatively associated with grey crescent (OR = 0.47, *p* = 0.01), neural rim notching (OR = 0.43, *p* = 0.01), and cylindrical (OR = 0.55, *p* < 0.001) or bean pot cup shapes (OR = 0.59, *p* < 0.001). Larger beta-PPA area was linked to older age (*p* = 0.008) and large vertical CDR (*p* = 0.03), but was less likely with conus pigmentosus (*p* = 0.001), deeper cup depth (*p* = 0.006), higher IOP (*p* = 0.005), and rounder discs (*p* = 0.01). Higher polygenic risk score (*p* = 0.007) and presence of the SNP rs34957764 in ROCK1P1 (*p* = 0.053) were associated with beta-PPA, but the same SNP was also associated with lesser area of beta-PPA (*p* = 0.03). Regions of beta-PPA correlated with greater RNFL loss (*p* < 0.01).

**Conclusions:**

Beta-PPA is strongly associated with older age and high CDR among individuals with POAG. These regions are vulnerable to RNFL loss.

## Introduction

Peripapillary Atrophy (PPA) is a morphologic change to the optic nerve head characterised by atrophy of the retinal pigment epithelium, photoreceptors, and choriocapillaris, resulting in visible choroidal vessels and sclera [1–3]. PPA existing with Bruch’s membrane (BM) is termed beta-zone Peripapillary Atrophy (beta-PPA) and is associated with primary open-angle glaucoma (POAG), while PPA without overlying BM is termed gamma-PPA and is associated with myopia [4–6].

Though the association between beta-PPA and POAG has been studied for almost three decades, results on this association remain mixed. As early as 1996, Park et al. described the association between PPA and progressive optic disc damage and visual field loss in glaucoma [7]. This finding has been substantiated several times since, and beta-PPA has been well-described as a risk factor for POAG-related visual field loss [8–11]. Several studies have also described an association between retinal nerve fibre layer (RNFL) thinning and beta-PPA, including one study from our group [12–15]. Furthermore, evidence suggests that beta-PPA location correlates spatially with the region of most rapid future visual field loss progression in patients with POAG, and that beta-PPA irregularity correlates with visual field loss progression [13, 16].

Some groups have suggested that beta-PPA may be used as a diagnostic marker for POAG [10]. To improve the diagnostic ability of beta-PPA, however, a more precise understanding of beta-PPA characteristics is needed, and several limitations need to be overcome. For example, some studies have found no association between beta-PPA and glaucoma, while others have described the need for more sensitive screening tools that can also accommodate ethnic differences in peripapillary structures and background retinal pigmentation [10, 17, 18]. This follows with POAG in general, which remains particularly understudied in individuals of African ancestry—despite having the highest incidence and most severe progression and outcomes of the disease, as well as a higher prevalence of beta-PPA [18–22]. Other risk factors associated with an increased area of beta-PPA are also yet to be elucidated. For these reasons and to better understand the properties of beta-PPA, population-based studies of beta-PPA demographics, including prevalence, risk factors, and associations with disease, are needed, especially in understudied populations.

In this study, we investigated the demographic, qualitative disc, ocular, and genetic risk factors associated with the presence and area of beta-PPA among POAG cases of African ancestry. Eligible POAG cases from the Primary Open-Angle African American Glaucoma Genetics (POAAGG) study were included in analyses. We evaluated the prevalence of beta-PPA in this African ancestry cohort and investigated the association of its presence and area (i.e., ratio of beta-PPA area to disc area) with demographic, qualitative disc, ocular, and genetic risk factors.

## Methodology

### Study population

Subjects included in this study were part of the larger POAAGG study. POAAGG subjects were 35 year or older and self-identified as being of African ancestry or admixed African ancestry (i.e., Black, African American, or African Caribbean). Subjects were recruited during regularly scheduled ophthalmology appointments at the University of Pennsylvania and several external sites in the Philadelphia, PA region between 2010 and 2019. Each subject provided a genomic DNA sample and completed an interview and ophthalmic exam, whereby an ophthalmologist or fellowship-trained glaucoma specialist classified each patient as a case, control, or suspect based on detailed clinical criteria. These details, as well as inclusion criteria, exclusion criteria, and phenotyping methods for the POAAGG study, are extensively described elsewhere [23]. Informed consent was obtained for all subjects, Institutional Review Board (IRB)/Ethics Committee approval was obtained by the University of Pennsylvania IRB, and the research adhered to the tenets of the Declaration of Helsinki.

### Demographic, behavioural, and systemic data collection

Demographic, behavioural, and systemic disease information was collected from each subject using an enrolment questionnaire, during a standardised enrolment interview, and finally supplemented through electronic medical records.

### Ocular data collection

Glaucoma-related phenotypes, including visual acuity (VA), cup-to-disc ratio (CDR), intraocular pressure (IOP), central corneal thickness (CCT), pattern standard deviation (PSD), and RNFL thickness, were collected from POAG cases during an ophthalmic exam. Average RNFL thickness was collected through optical coherence topography (OCT) using Cirrus OCT (Carl Zeiss Meditec, Dublin, CA). Only phenotypes with images taken within 6 months of the study date were used in this analysis.

Qualitative features of the optic cup and disc, including presence of beta-PPA, were evaluated using 30° colour stereo disc photos taken using the Topcon TRC 50EX retinal camera (Topcon Corp. of America, Paramus, New Jersey, USA). As described in detail elsewhere, three non-physician graders were trained by glaucoma specialists to grade these images using a stereo viewer (Screen-Vu stereoscope, Portland, Oregon, USA) [24]. Two graders independently completed a standardised grading of each photo. Cup depth was categorised as shallow, moderate, or deep by each grader. Discrepancies between the two graders were adjudicated by the Reading Center Director [24].

Quantitative analysis of beta-PPA area—which provides an objective measure of beta-PPA progression and is significantly associated with a diagnosis of glaucoma—followed a similar protocol [25]. Two graders independently completed a standardised grading of each disc photo using the ImageJ software (National Institutes of Health, Bethesda, Maryland, USA) to outline concentric regions around the optic nerve—including the edge of the optic disc, scleral ring, and beta-PPA—using a freehand selection tool. The beta-PPA zone was defined as the area adjacent to the scleral ring, characterised by the absence of the retinal pigment epithelium and exposure of the underlying sclera or large choroidal vessels. The ratio of beta-PPA area to optic nerve area was then calculated. Graders ensured consistency in measurement by adhering to calibration steps in ImageJ, setting the pixel-to-micrometre scale, and including parameters such as area, perimeter, and centroid coordinates in the analysis. A previous publication from our group demonstrated strong inter-grader reliability in measuring the optic cup and disc on these images [24]. An adjudication process for quantitative grading that utilises dice coefficients is detailed elsewhere [26].

### Genetic data collection

A prior genome-wide association study (GWAS) on cases and controls in the POAAGG cohort identified three single nucleotide polymorphisms (SNPs) as likely causal of POAG [27]. These variants were selected to assess for their association with beta-PPA in this study. The variants are: rs1666698 mapping to *DBF4P2* on chromosome 2, rs11824032 mapping to *ARHGEF12* on chromosome 11, and rs34957764 mapping to *ROCK1P1* on chromosome 18. Further, our group previously developed a polygenic risk score (PRS) and genetic risk score (GRS) for development of POAG individuals of African ancestry, which was also assessed in this study for association with beta-PPA [27]. The PRS considers the cumulative effect of many genetic variants on POAG, while the GRS focuses on a smaller number of variants with a stronger association with POAG. We also assessed ancestry using FastSTRUCTURE, Admixture, and Plink software among autosomal genotypes in POAAGG, generating ancestral components q0 and q1, representing African and European ancestral components, respectively. Lower values of q0 denote higher degrees of African ancestry and lower degrees of European ancestry. Among POAG cases, the mean African ancestry proportion (q0) was 0.25 (SD 0.19), corresponding to an average European ancestry of 0.75. Among controls, the mean q0 was 0.27 (SD 0.18). These values are consistent with the admixed nature of the cohort and align with findings from prior ancestry analyses in the POAAGG study [28].

### Statistical analysis

Descriptive analyses were performed using mean, standard deviation (SD) for continuous measures, and count and percentage for categorical measures. Comparison of demographic and ocular characteristics between eyes with versus without beta-PPA was performed using generalised linear models to account for the inter-eye correlation among participants with both eyes eligible for the analysis, the benefits of which are extensively discussed elsewhere [29–33]. Risk factors associated with presence of beta-PPA were evaluated using univariable and multivariable logistic regression models, and risk factors associated with area of beta-PPA were evaluated using univariable and multivariable linear regression models. The multivariable model started with including all factors with *p* < 0.20 in univariable analysis and went through backward variable selection by only keeping those factors with *p* < 0.05 in the final multivariable model. A backward selection model was used to avoid overfitting of the multivariable model, which could distort any associations. All the statistical analyses were performed in SAS V.9.4 (SAS Institute Inc., Cary, NC), and two-sided *p* < 0.05 was considered to be statistically significant. The datasets generated during and/or analysed during the current study are available from the corresponding author on reasonable request.

## Results

A total of 3381 eyes of POAG cases were included in this study, of which 969 (28.6%) had beta-PPA. The univariable analysis results for the association of demographic, qualitative disc, and clinical risk factors with the presence of beta-PPA are reported in Supplemental Tables 1, 2, and 3, respectively.

Results from multivariate analysis for risk factors associated with presence of beta-PPA are shown in Table 1. Older age (*p* < 0.001), larger vertical CDR (*p* = 0.007) were associated with higher risk of beta-PPA, while female gender (*p* < 0.001), pigmentation within the disc margins termed gray crescent (*p* = 0.01), notching of the neural rim (*p* = 0.01), and cylindrical or bean pot cup shape (*p* < 0.001) were associated with lower risk of beta-PPA.

**Table 1:** Multivariable analysis for risk factor of presence of beta-PPA (cases *N* = 969, controls *N* = 125).

		Odds ratio (95% CI)	*P* value
Gender	Male	Ref	<0.001
	Female	0.53 (0.39, 0.72)	
Age group	≤60	Ref	<0.001
	(60,75]	1.92 (1.25, 2.93)	
	≥75	2.90 (1.81, 4.64)	
Gray crescent	No	Ref	0.01
	Yes	0.47 (0.26, 0.84)	
Notching of neural rim	No	Ref	0.01
	Yes	0.43 (0.22, 0.84)	
Shape of cup	Conical	Ref	<0.001
	Cylindrical	0.55 (0.40, 0.75)	
	Partial Bean Pot and Bean Pot	0.59 (0.36, 0.95)	
Vertical cup-to-disc ratio group	≤0.5	Ref	0.007
	(0.5, 0.7]	0.96 (0.60, 1.52)	
	(0.7, 1]	1.58 (0.99, 2.52)	
Refractive Error (per 0.1 units increase)	Mean (SD)	0.987 (0.980, 0.994)	<0.001

Multivariable analysis for risk factors of presence of beta-PPA among those with and without beta-PPA.

The univariable analysis results for the risk factors associated with the area of beta-PPA are summarised in Supplemental Tables 4, 5, and 6.

Results from multivariate analysis for risk factors associated with area of beta-PPA are shown in Table 2. Greater area of beta-PPA was more likely in eyes of older age (*p* = 0.008) and with a larger vertical CDR (*p* = 0.03), and less likely in eyes with pigmentation outside the optic disc margins, termed conus pigmentosus (*p* = 0.001), deep cup depth (*p* = 0.006), high IOP (*p* = 0.005), and increased roundness of disc (*p* = 0.01).

**Table 2:** Multivariable analysis for risk factor of proportion of beta-PPA to Disc (*N* = 744 eye cases).

		Lease square mean difference (95% CI)	*P* value
Age group	≤60	Ref	0.008
	(60, 75]	0.10 (−0.08, 0.29)	
	≥75	0.21 (0.08, 0.34)	
Conus pigmentosus	No	Ref	0.001
	Yes	−0.16 (−0.27, −0.06)	
Cup Depth	Shallow	Ref	0.006
	Moderate	−0.25 (−0.62,0.12)	
	Deep	−0.38 (−0.77, 0.01)	
Highest intraocular pressure (per 1 unit increase)	per 1 unit increase	−0.02 (−0.03, −0.01)	0.005
Notching of neural rim	No	Ref	0.06
	Yes	−0.10 (−0.21, 0.01)	
Roundness of disc (per 0.1 units increase)	per 1 unit increase	−0.29 (−0.52, −0.06)	0.01
Vertical cup-to-disc ratio group	≤0.5	Ref	0.03
	(0.5, 0.7]	0.17 (0.04, 0.30)	
	(0.7, 1]	0.24 (0.03, 0.46)	

Multivariable analysis for risk factors of proportion of beta-PPA among those with beta-PPA. Eyes with missing data in any of the predictors in the multivariable model were excluded from this analysis.

The univariable analysis results for genetic risk factors associated with the presence and area of beta-PPA are shown in Tables 3 and 4, respectively. Higher PRS (*p* = 0.007) and presence of one rs34957764 SNP allele (*p* = 0.053) were both more likely in eyes with beta-PPA. However, we also found that larger beta-PPA area was less likely given the presence of one rs34957764 SNP allele (*p* = 0.03).

**Table 3:** Univariable analysis for genetic risk factor of presence of beta-PPA (Cases).

		Presence of beta-PPA		
		No [*N* = 2412 eyes (%)]	Yes [*N* = 969 eyes (%)]	*P* value
q1	*N*	1424	614	0.13
	Mean (SD)	0.75 (0.19)	0.73 (0.20)	
Genetic risk score	*N*	2109	875	0.06
	Mean (SD)	1.44 (0.49)	1.40 (0.48)	
Polygenic risk score	*N*	2109	875	0.007
	Mean (SD)	51.90 (0.77)	52.00 (0.79)	
(rs1666698_ALT/DBF4P2) chr2:111570643:T:C	0 alleles	91 (4.3%)	49 (5.6%)	0.14
	1 allele	885 (42.0%)	330 (37.7%)	
	2 alleles	1133 (53.7%)	496 (56.7%)	
(rs11824032_ALT/ARHGEF12) chr11:120354080:G:A_G	0 alleles	1127 (53.4%)	470 (53.7%)	0.94
	1 allele	804 (38.1%)	335 (38.3%)	
	2 alleles	178 (8.4%)	70 (8.0%)	
(rs34957764_ALT/ROCK1P1) chr18:93327:C:T_C	0 alleles	1933 (91.7%)	778 (88.9%)	0.053
	1 allele	176 (8.3%)	97 (11.1%)	

Univariable analysis for genetic risk factors for presence of beta-PPA. q1 represents the degree of African ancestry. The genetic and polygenic risk scores were developed by our group to predict the likelihood of developing POAG. GRS and PRS were analysed as raw scores for consistency with prior POAAGG studies. Standardized (z-score) versions of these scores yielded similar results and are available upon request. The 3 SNPS were identified by a large GWAS study as likely causal of POAG.

**Table 4:** Univariable analysis for genetic risk factor of proportion of beta-PPA to Disc (Cases).

		*N*	Proportion of beta-PPA changes (95% CI)	*P* value
q1 (per 0.1 units		954	−0.01 (−0.03, 0.02)	0.57
Genetic risk score (per 1 unit increase		954	0.06 (−0.10, 0.21	0.47
Polygenic risk score (per 1 unit increase)		954	−0.01 (−0.08, 0.06)	0.77
	**Alleles present**	***N***	**Mean (SD)**	***P*** **value**
(rs1666698_ALT/DBF4P2) chr2:111570643:T:C	0	46	0.51 (0.98)	0.37
	1	325	0.48 (1.00)	
	2	489	0.38 (0.47)	
(rs11824032_ALT/ARHGEF12) chr11:120354080:G:A_G	0	462	0.41 (0.86)	0.92
	1	330	0.43 (0.56)	
	2	68	0.46 (0.71)	
(rs34957764_ALT/ROCK1P1) chr18:93327:C:T_C	0	767	0.43 (0.78)	0.03
	1	93	0.32 (0.39)	

Univariable analysis for genetic risk factors of proportion of beta-PPA. q1 represents the degree of African ancestry. The genetic and polygenic risk scores were developed by our group to predict the likelihood of developing POAG. GRS and PRS were analysed as raw scores for consistency with prior POAAGG studies. Standardised (z-score) versions of these scores yielded similar results and are available upon request. The 3 SNPS were identified by a large GWAS study as likely causal of POAG.

Since thinning of the retinal nerve fibre layer (RNFL) is a feature of POAG, RNFL thickness was compared between regions of beta-PPA and regions without beta-PPA in the same eye. Additionally, the RNFL thickness for regions of beta-PPA was compared to the RNFL thickness of eyes that do not contain any beta-PPA (Table 5). Significant RNFL thinning was found in eye regions with beta-PPA in both analyses (*p* = 0.0095 and *p* = 0.0044, respectively).

**Table 5:** Retinal nerve fiber layer (RNFL) thickness in eye regions that do and do not contain beta-PPA.

	*N* (eyes)	Mean RNFL	*P* value
Eyes regions with beta-PPA	83	67.26	
Eye region without beta-PPA in eyes with some beta-PPA (difference: regions with beta-PPA – regions without beta-PPA)	78	72.16 (−4.90)	0.0095
Eyes without any beta-PPA (difference: regions with beta-PPA – regions without beta-PPA)	302	72.83 (−5.57)	0.004

Retinal nerve fibre layer (RNFL) thickness in eye regions that do and do not contain beta-PPA.

## Discussion

In this study, we investigated the risk factors associated with the presence of beta-PPA in a large African-ancestry cohort with POAG. We showed that among individuals of African ancestry with POAG, beta-PPA is strongly associated with both older age and high vertical CDR—risk factors and manifestations of glaucoma. This is in accordance with the findings by another study that reported a correlation of beta zone area with age and glaucoma in a Japanese population [34]. The shared risk factors between beta-PPA and POAG support the hypothesis that an interplay exists between the two. However, while beta-PPA has been proposed as a potential marker of POAG development, our data are limited to individuals already diagnosed with POAG and cannot address whether beta-PPA precedes or results from the disease. We further showed a negative association between presence of beta-PPA and female gender, presence of grey crescent, notching of neural rim, and non-conical shape of cup. To the best of our knowledge, only one study to date has examined associations between beta-PPA and any of these factors—a study by our group which found the presence of grey crescent to be a risk factor for beta-PPA [35].

More research is needed to explore the conditions by which beta-PPA and POAG interplay. One study found that 10–20% of normally aging eyes present with beta-PPA [36]. Ten-year follow-up in this study indicated that the growth of beta-PPA in non-POAG eyes morphologically differed from the spread of beta-PPA in POAG eyes. Another study found that beta-PPA was associated with worse progression in glaucomatous eyes of European descent compared to African descent [18]. In this study, univariable analysis showed that the presence of beta-PPA was associated with higher likelihood of past glaucoma surgery, which can be indicative of more severe or rapidly progressing disease. Additionally, our study found the prevalence of beta-PPA in POAG equal to 0.29, while previous meta-analyses found the prevalence of beta-PPA in OAG equal to 0.73 [10]. While this may be explained by the association of African ancestry with younger onset of POAG, these variable findings suggest that additional studies are needed to understand the risk factors for beta-PPA coinciding with glaucomatous progression.

While prior research suggests that there is a strong genetic component of beta-PPA, to our knowledge, no other study has identified specific genetic or ancestral associations with beta-PPA [18, 37]. We did not find a clear association between beta-PPA and the three SNPs previously implicated in a large GWAS of POAG. However, we found that a higher PRS—previously developed and validated in our POAAGG cohort to predict POAG risk—was significantly associated with presence of beta-PPA [27]. This finding supports a growing body of evidence that genetic predisposition to POAG may also influence optic nerve head structural features such as beta-PPA, further linking genetic risk burden to clinical phenotypes of glaucomatous damage [27]. This aligns with our prior work in the POAAGG cohort, justifying the use of a genetic risk score to stratify POAG risk in African ancestry individuals, as described in our development of a PRS model tailored for this population [27].

Finally, we also showed that eye regions with beta-PPA demonstrate greater RNFL thinning compared to eye regions without beta-PPA. This finding has been corroborated elsewhere in the literature, whereby the spatial extent of beta-PPA corresponded to the spatial extent of RNFL thinning—in the classic ISNT (inferior, superior, nasal, temporal) pattern that describes glaucomatous RNFL thinning [12, 36, 38–41]. As such, these findings support the conclusion that beta-PPA may be an aspect of glaucomatous change.

Our study has several limitations. One such limitation is the yet uncertain clinical utility of beta-PPA in POAG. Several studies have failed to report an association of beta-PPA with POAG, while others have commented on the little incremental clinical value added by assessing PPA in addition to the age, CCT, IOP, and CDR evaluations that comprise a typical glaucoma evaluation [17, 42]. On this note, a key limitation of our study is that it includes only individuals with POAG and does not incorporate a control group without glaucoma. Therefore, while we can evaluate correlates of beta-PPA expression within POAG, we cannot infer whether beta-PPA is predictive of POAG onset or progression in the broader population. Methodologically, while optic nerve parameters were graded by trained researchers with a clear adjudication process, subjective grading could have impacted data collection. Additionally, subjects without or with low-quality images were excluded, introducing potential for selection bias. Because our genetic analyses were restricted to individuals with POAG, we cannot determine whether SNP associations are specific to beta-PPA or reflect broader genetic risk for glaucoma. Future studies incorporating glaucoma-free controls with and without beta-PPA are needed to clarify this distinction. Finally, the generalisability of this study suffers from our exclusive focus on individuals of African ancestry with POAG. However, we chose to focus on this group in order to address the disparities faced by this vastly understudied population in terms of prevalence of the condition and vision loss. Expanding our study to include individuals of African ancestry without POAG would also yield stronger results for our study.

In conclusion, we found that beta-PPA is associated with older age and increased vertical CDR in a population of African ancestry individuals with POAG. We failed to identify clear genetic associations with beta-PPA, but did find that beta-PPA is associated with loss of RNFL. Further research is needed to elucidate the clinical utility of beta-PPA in predicting glaucomatous progression, as well as the specific characteristics of beta-PPA that indicate glaucomatous degeneration.

## Summary

### What was known before

- Glaucoma Association: Beta-peripapillary atrophy (beta-PPA) is a recognised morphologic change at the optic nerve head that is linked to glaucomatous damage such as optic disc deterioration, visual field loss, and retinal nerve fibre layer (RNFL) thinning. Research Gap in Risk Factors: No previous studies have comprehensively investigated the demographic, ocular, or genetic risk factors for beta-PPA across any ethnicity, marking an important gap in our understanding of its development.

### What this study adds

- Risk Factor Insights: In a large cohort of individuals of African ancestry with primary open-angle glaucoma, beta-PPA was present in 28.6% of eyes and was strongly associated with older age and larger vertical cup-to-disc ratio, reinforcing its role as a glaucomatous marker in a high-risk population. Distinct Optic Nerve Head Characteristics: The study found that beta-PPA was significantly less common in female eyes and in those exhibiting features such as a grey crescent, neural rim notching, or non-conical cup shapes. These findings highlight unique optic nerve head structural differences that may influence beta-PPA development. Complex Genetic and Structural Associations: While the rs34957764 SNP was linked to the presence of beta-PPA, it paradoxically correlated with a smaller beta-PPA area. Additionally, regions with beta-PPA corresponded with significant RNFL thinning, underlining its clinical relevance in glaucoma progression.

## Supplementary information

Supplemental Table 1

Supplemental Table 2

Supplemental Table 3

Supplemental Table 4

Supplemental Table 5

Supplemental Table 6
