# Supplementary material for: Risk factors associated with beta-peripapillary atrophy in individuals of African ancestry with primary open-angle glaucoma
Source: Eye (Lond). 2025 Oct 7;39(17):3180–6. doi: 10.1038/s41433-025-03988-8 (PMC12623487; doi:10.1038/s41433-025-03988-8)
Supplement: Supplementary file 1 — Supplemental Table 1 [file 41433_2025_3988_MOESM1_ESM.pdf]

Supplemental Table 1. Univariable Analysis for Demographic Risk Factor of Presence of Beta-PPA (Cases)			
	**Presence of beta-PPA**		
	**No [N=2412 eyes (%)]**	**Yes [N=969 eyes (%)]**	**P-value**
Age at Enrollment			
Mean (SD)	68.0 (11.4)	72.1 (10.9)	<0.001
Median (Q1, Q3)	69.0 (60.0,77.0)	72.0 (65.0,81.0)	.
Min, Max	35.0,103.0	40.0,103.0	
N	2412	969	
Age group			
<= 60	645 (26.7%)	144 (14.9%)	<0.001
(60,75]	1096 (45.4%)	434 (44.8%)	
>=75	671 (27.8%)	391 (40.4%)	
Gender			
Male	956 (39.6%)	437 (45.1%)	0.02
Female	1456 (60.4%)	532 (54.9%)	
Body Mass Index (BMI)			
Mean (SD)	30.2 (6.6)	28.9 (6.3)	<0.001
Median (Q1, Q3)	29.3 (25.6,34.1)	28.1 (24.5,32.3)	.
Min, Max	0.0,59.6	15.3,56.2	
N	2362	953	
Has patient been diagnosed with diabetes?			
No	1404 (59.2%)	599 (62.5%)	0.15
Yes	968 (40.8%)	359 (37.5%)	
Has patient been diagnosed with hypertension?			
No	520 (21.9%)	199 (20.7%)	0.54
Yes	1854 (78.1%)	761 (79.3%)	
Family history of glaucoma?			
No	1072 (44.4%)	481 (49.6%)	0.03
Yes	1340 (55.6%)	488 (50.4%)	
History of Alcohol Use?			

Supplemental Table 1. Univariable Analysis for Demographic Risk Factor of Presence of Beta-PPA (Cases)			
	**Presence of beta-PPA**		
	**No [N=2412 eyes (%)]**	**Yes [N=969 eyes (%)]**	**P-value**
No	1220 (52.2%)	535 (56.4%)	0.07
Yes	1115 (47.8%)	413 (43.6%)	
History of Tobacco Use?			
No	1095 (46.8%)	413 (43.4%)	0.15
Yes	1244 (53.2%)	538 (56.6%)	
Glaucoma Surgery?			
No	1739 (73.6%)	638 (66.9%)	0.001
Yes	625 (26.4%)	316 (33.1%)	
Previous Cataract Surgery?			
No	2381 (98.7%)	958 (98.9%)	0.74
Yes	31 (1.3%)	11 (1.1%)	
Univariable Analysis for Demographic Risk Factor of Presence of Beta-PPA			
