# Supplementary material for: Risk factors associated with beta-peripapillary atrophy in individuals of African ancestry with primary open-angle glaucoma
Source: Eye (Lond). 2025 Oct 7;39(17):3180–6. doi: 10.1038/s41433-025-03988-8 (PMC12623487; doi:10.1038/s41433-025-03988-8)
Supplement: Supplementary file 2 — Supplemental Table 2 [file 41433_2025_3988_MOESM2_ESM.pdf]

Supplemental Table 2. Univariable Analysis for Qualitative Disc Risk Factor of Presence of Beta-PPA (Cases)			
	**Presence of beta-PPA**		
	**No [N=2412 eyes (%)]**	**Yes [N=969 eyes (%)]**	**P-value**
Disc shape			
Round	1025 (42.6%)	419 (43.5%)	0.69
Oval	1379 (57.4%)	545 (56.5%)	
Shape of cup			
Conical	876 (37.9%)	458 (49.3%)	<0.001
Cylindrical	1104 (47.8%)	357 (38.4%)	
Partial Bean Pot and Bean Pot	332 (14.4%)	114 (12.3%)	
Cup Depth			
Shallow	239 (10.3%)	137 (14.6%)	<0.001
Moderate	1448 (62.5%)	624 (66.3%)	
Deep	628 (27.1%)	180 (19.1%)	
Stereoscopically diagnosed Tilted Disc			
No	1872 (80.8%)	750 (79.7%)	0.50
Yes	444 (19.2%)	191 (20.3%)	
Disc hemorrhage			
No	2365 (98.1%)	955 (98.8%)	0.16
Yes	47 (1.9%)	12 (1.2%)	
Arteriole narrowing			
No	2381 (98.8%)	942 (97.6%)	0.01
Yes	29 (1.2%)	23 (2.4%)	
Venule narrowing			
No	2383 (98.8%)	944 (97.7%)	0.02
Yes	28 (1.2%)	22 (2.3%)	
Visible pores of the lamina cribrosa			
No	569 (28.7%)	225 (26.5%)	0.30
Yes	1416 (71.3%)	624 (73.5%)	

Supplemental Table 2. Univariable Analysis for Qualitative Disc Risk Factor of Presence of Beta-PPA (Cases)			
	**Presence of beta-PPA**		
	**No [N=2412 eyes (%)]**	**Yes [N=969 eyes (%)]**	**P-value**
Baring of the circumlinear vessels			
No	1778 (74.4%)	740 (77.2%)	0.10
Yes	611 (25.6%)	218 (22.8%)	
Vessels overpass			
No	2292 (98.8%)	923 (98.1%)	0.10
Yes	27 (1.2%)	18 (1.9%)	
Bayonetting			
No	1460 (62.8%)	661 (70.1%)	<0.001
Moderate	707 (30.4%)	240 (25.5%)	
Severe	158 (6.8%)	42 (4.5%)	
Nasalization of the vessels			
No	1474 (61.3%)	613 (63.5%)	0.29
Yes	932 (38.7%)	353 (36.5%)	
Gray crescent			
No	2162 (89.7%)	918 (94.7%)	<0.001
Yes	249 (10.3%)	51 (5.3%)	
Conus pigmentosus			
No	2235 (92.7%)	900 (93.0%)	0.79
Yes	176 (7.3%)	68 (7.0%)	
Notching of neural rim			
No	2192 (93.3%)	911 (95.5%)	0.02
Yes	158 (6.7%)	43 (4.5%)	
Pallor of the neural rim			
No	2309 (95.7%)	925 (95.8%)	0.97
Yes	103 (4.3%)	41 (4.2%)	
Vertical cup-to-disc ratio (CDR)			

Supplemental Table 2. Univariable Analysis for Qualitative Disc Risk Factor of Presence of Beta-PPA (Cases)			
	**Presence of beta-PPA**		
	**No [N=2412 eyes (%)]**	**Yes [N=969 eyes (%)]**	**P-value**
Mean (SD)	0.69 (0.17)	0.74 (0.17)	<0.001
Median (Q1, Q3)	0.71 (0.60,0.82)	0.76 (0.65,0.86)	
Min, Max	0.10,1.00	0.10,1.00	
Vertical CDR			
<=0.5	240 (16.6%)	98 (12.9%)	<0.001
(0.5,0.7]	476 (33.0%)	192 (25.2%)	
(0.7,1]	726 (50.3%)	472 (61.9%)	
Circularity of disc			
Mean (SD)	0.958 (0.024)	0.951 (0.026)	<0.001
Median (Q1, Q3)	0.960 (0.951,0.970)	0.955 (0.945,0.963)	<0.001
Min, Max	0.505,0.985	0.660,0.985	.
N	2400	960	.
Roundness of disc			
Mean (SD)	0.884 (0.054)	0.874 (0.063)	<0.001
Median (Q1, Q3)	0.890 (0.850,0.925)	0.885 (0.840,0.920)	0.001
Min, Max	0.530,0.990	0.560,0.985	.
N	2400	960	.
Univariable analysis for qualitative disc risk factors for the presence of beta-PPA			
