# Supplementary material for: Risk factors associated with beta-peripapillary atrophy in individuals of African ancestry with primary open-angle glaucoma
Source: Eye (Lond). 2025 Oct 7;39(17):3180–6. doi: 10.1038/s41433-025-03988-8 (PMC12623487; doi:10.1038/s41433-025-03988-8)
Supplement: Supplementary file 3 — Supplemental Table 3 [file 41433_2025_3988_MOESM3_ESM.pdf]

Supplemental Table 3. Univariable Analysis for Clinical Ocular Features of Presence of Beta-PPA (Cases)			
	**Presence of beta-PPA**		
	**No [N=2412 eyes (%)]**	**Yes [N=969 eyes (%)]**	**P-value**
Refractive Error			
Mean (SD)	0.002 (2.862)	-0.699 (2.860)	<0.001
Median (Q1, Q3)	0.000 (-1.000,1.125)	-0.250 (-1.625,0.875)	.
Min, Max	-9.750,75.500	-37.750,4.750	.
N	1337	473	.
Highest intraocular pressure			
Mean (SD)	11.38 (3.30)	11.09 (3.64)	0.0504
Median (Q1, Q3)	11.00 (10.00,13.00)	11.00 (9.00,13.00)	.
Min, Max	1.00,36.00	1.00,60.00	.
N	2410	969	.
Central Corneal Thickness			
Mean (SD)	534.96 (38.73)	534.87 (41.64)	0.98
Median (Q1, Q3)	534.00 (510.00,561.00)	530.00 (509.00,560.00)	.
Min, Max	422.00,657.00	420.00,690.00	.
N	987	422	.
Cup Disc Ratio			
Mean (SD)	0.70 (0.17)	0.74 (0.19)	0.003
Median (Q1, Q3)	0.72 (0.60,0.85)	0.80 (0.65,0.90)	.
Min, Max	0.10,1.00	0.10,1.00	.
N	1080	426	.
Visual Acuity			
Mean (SD)	0.22 (0.38)	0.36 (0.59)	<0.001
Median (Q1, Q3)	0.10 (0.00,0.30)	0.18 (0.00,0.40)	.
Min, Max	-0.12,3.00	-0.12,3.00	.
N	981	425	.
Nerve Fiber Layer Thickness			
Mean (SD)	75.51 (14.68)	72.57 (13.49)	0.02
Median (Q1, Q3)	76.00 (64.00,86.00)	73.00 (63.00,81.00)	.
Min, Max	31.00,120.00	43.00,119.00	.

Supplemental Table 3. Univariable Analysis for Clinical Ocular Features of Presence of Beta-PPA (Cases)			
	**Presence of beta-PPA**		
	**No [N=2412 eyes (%)]**	**Yes [N=969 eyes (%)]**	**P-value**
N	472	211	.
Visual Field			
Mean (SD)	-7.07 (8.76)	-9.30 (10.12)	0.01
Median (Q1, Q3)	-3.70 (-9.65,-1.10)	-5.42 (-16.23,-2.37)	.
Min, Max	-33.15,8.96	-32.15,22.08	.
N	547	228	.
Univariable analysis for clinical ocular features for the presence of beta-PPA			
