# Supplementary material for: Risk factors associated with beta-peripapillary atrophy in individuals of African ancestry with primary open-angle glaucoma
Source: Eye (Lond). 2025 Oct 7;39(17):3180–6. doi: 10.1038/s41433-025-03988-8 (PMC12623487; doi:10.1038/s41433-025-03988-8)
Supplement: Supplementary file 4 — Supplemental Table 4 [file 41433_2025_3988_MOESM4_ESM.pdf]

Supplemental Table 4. Univariable Analysis for Demographic Risk Factor of Proportion of Beta-PPA to Disc (Cases)			
	**Glaucoma Cases (N = 969 eyes)**		
	**N**	**Mean (SD)**	**P value**
Age group			
<= 60	142	0.28 (0.46)	<0.001
(60,75]	426	0.40 (0.90)	
>=75	386	0.49 (0.56)	
Gender			
Male	430	0.44 (0.91)	0.51
Female	524	0.40 (0.52)	
Has patient been diagnosed with diabetes?			
No	590	0.41 (0.55)	0.92
Yes	353	0.42 (0.95)	
Has patient been diagnosed with hypertension			
No	194	0.40 (0.64)	0.69
Yes	751	0.42 (0.74)	
Family history of glaucoma?			
No	472	0.37 (0.45)	0.14
Yes	482	0.46 (0.91)	
History of Alcohol Use?			
No	526	0.42 (0.56)	0.86
Yes	407	0.43 (0.90)	
History of Tobacco Use?			
No	405	0.37 (0.44)	0.10
Yes	531	0.46 (0.89)	
Glaucoma Surgery?			
No	632	0.36 (0.43)	0.03
Yes	307	0.55 (1.10)	
Previous Cataract Surgery?			

Supplemental Table 4. Univariable Analysis for Demographic Risk Factor of Proportion of Beta-PPA to Disc (Cases)			
	**Glaucoma Cases (N = 969 eyes)**		
	**N**	**Mean (SD)**	**P value**
No	944	0.42 (0.73)	0.58
Yes	10	0.50 (0.46)	
Univariable analysis for demographic risk factors for the proportion of Beta-PPA to disc (area of beta-PPA)			
