# Supplementary material for: Risk factors associated with beta-peripapillary atrophy in individuals of African ancestry with primary open-angle glaucoma
Source: Eye (Lond). 2025 Oct 7;39(17):3180–6. doi: 10.1038/s41433-025-03988-8 (PMC12623487; doi:10.1038/s41433-025-03988-8)
Supplement: Supplementary file 5 — Supplemental Table 5 [file 41433_2025_3988_MOESM5_ESM.pdf]

Supplemental Table 5. Univariable Analysis for Qualitative Disc Characteristics Risk Factor of Proportion of Beta-PPA to Disc (Cases)			
	Glaucoma Cases (N = 969 eyes)		
	N	Mean (SD)	P-value
Disc shape			
Round	416	0.36 (0.48)	0.04
Oval	533	0.47 (0.87)	
Shape of cup			
Conical	450	0.50 (0.94)	0.01
Cylindrical	352	0.34 (0.43)	
Partial Bean Pot and Bean Pot	113	0.31 (0.29)	
Cup Depth			
Shallow	136	0.64 (1.44)	<0.001
Moderate	614	0.41 (0.52)	
Deep	178	0.28 (0.32)	
Stereoscopically diagnosed Tilted Disc			
No	739	0.43 (0.78)	0.28
Yes	188	0.37 (0.40)	
Disc hemorrhage			
No	941	0.42 (0.73)	0.91
Yes	12	0.44 (0.54)	
Arteriole narrowing			
No	930	0.42 (0.73)	0.71
Yes	22	0.46 (0.51)	
Venule narrowing			
No	930	0.42 (0.73)	0.68
Yes	22	0.39 (0.33)	
Visible pores of the lamina cribrosa			
No	222	0.50 (0.68)	0.13
Yes	616	0.40 (0.78)	

Supplemental Table 5. Univariable Analysis for Qualitative Disc Characteristics Risk Factor of Proportion of Beta-PPA to Disc (Cases)			
	Glaucoma Cases (N = 969 eyes)		
	N	Mean (SD)	P-value
Baring of the circumlinear vessels			
No	729	0.45 (0.79)	0.01
Yes	215	0.33 (0.46)	
Vessels overpass			
No	910	0.42 (0.72)	0.57
Yes	18	0.48 (0.50)	
Bayonetting			
No	652	0.46 (0.81)	0.005
Moderate	238	0.32 (0.41)	
Severe	40	0.29 (0.29)	
Nasalization of the vessels			
No	600	0.46 (0.86)	0.02
Yes	351	0.34 (0.36)	
Gray crescent			
No	904	0.43 (0.73)	0.06
Yes	50	0.29 (0.46)	
Conus pigmentosus			
No	886	0.43 (0.74)	<0.001
Yes	67	0.26 (0.34)	
Notching of neural rim			
No	898	0.43 (0.74)	0.02
Yes	41	0.31 (0.26)	
Pallor of the neural rim			
No	910	0.42 (0.73)	0.65
Yes	41	0.39 (0.43)	
Vertical cup-to-disc ratio group			

**Supplemental Table 5. Univariable Analysis for Qualitative Disc Characteristics Risk Factor of Proportion of Beta-PPA to Disc (Cases)**			
	**Glaucoma Cases (N = 969 eyes)**		
	**N**	**Mean (SD)**	**P-value**
<=0.5	98	0.34 (0.35)	0.11
(0.5,0.7]	192	0.43 (0.61)	
(0.7,1]	467	0.46 (0.90)	
Univariable analysis for qualitative disc characteristics risk factors for the proportion of beta-PPA to disc (area of beta-PPA)			
