# Supplementary material for: Risk factors associated with beta-peripapillary atrophy in individuals of African ancestry with primary open-angle glaucoma
Source: Eye (Lond). 2025 Oct 7;39(17):3180–6. doi: 10.1038/s41433-025-03988-8 (PMC12623487; doi:10.1038/s41433-025-03988-8)
Supplement: Supplementary file 6 — Supplemental Table 6 [file 41433_2025_3988_MOESM6_ESM.pdf]

Supplemental Table 6. Univariable Analysis for Clinical Ocular Features of Proportion of Beta-PPA to Disc (Cases)			
	**Glaucoma Cases (N = 969 eyes)**		
	**N**	**Change of proportion of beta-PPA (95% CI)**	**p-value**
Refractive Error (per 0.1 unit increase)			
	728	-0.0005 (-0.0020,0.0009)	0.47
Highest intraocular pressure (per 1 unit increase)			
	954	-0.01 (-0.02,0.00)	0.06
Central Corneal Thickness (per 1 unit increase)			
	954	-0.0014 (-0.0033,0.0005)	0.14
Cup Disc Ratio (per 0.1 unit increase)			
	954	0.02 (-0.01,0.05)	0.16
Visual Acuity in logMAR (per 0.1 unit increase)			
	954	0.02 (0.01,0.03)	0.003
Nerve Fiber Layer Thickness (per 1 unit increase)			
	954	0.0050 (-0.0050,0.0150)	0.33
Visual Field (per 1 unit increase)			
	954	-0.0083 (-0.0151,-0.0015)	0.02
Univariable analysis for clinical ocular features for the proportion of beta-PPA to disc (area of beta-PPA)			
